# Editorial: Photosynthesis under fluctuating light

**DOI:** 10.3389/fpls.2023.1220360

**Published:** 2023-06-09

**Authors:** Fiamma Longoni, Michele Grieco, Stefano Santabarbara, Jeremy Harbinson

**Affiliations:** ^1^ Laboratory of Plant Physiology, Institute of Biology, University of Neuchâtel, Neuchâtel, Switzerland; ^2^ infarm - Indoor Urban Farming B.V., Berlin, Germany; ^3^ Photosynthesis Research Unit, Institute of Agricultural Biology and Biotechnology, National Research Council (CNR), Milan, Italy; ^4^ Wageningen University and Research, Wageningen, Netherlands

**Keywords:** light fluctuation, photosynthesis, combined abiotic stress, light harvesting, plant metabolic regulation

Photosynthetic organisms have colonized a wide variety of habitats in the terrestrial, freshwater and marine biomes. These habitats are characterized by significant differences in key physiological factors, including light intensity and spectral distribution, nutrients and water availability, temperature. The result is a conspicuously complex process that depends on the coordinated activity of several sub-processes whose adaptation underpins the optimisation of the photosynthetic apparatus to the specific habitat. The limited range of techniques available to measure photosynthesis in the field provides only partial insights into the operation and regulation of photosynthesis. The investigation of the diversity of adaptive strategies that have evolved as part of the adaptation of photosynthesis has largely been restricted to laboratory studies made on organisms grown and measured under stable, controlled conditions. This was, and still is, necessary in order to design reproducible, straightforward experiments addressing the fundamental mechanisms underlying the function and regulation of photosynthesis that, however, still eludes complete understanding even under the relatively controlled growth conditions.

Nonetheless, in recent years, there has been a growing interest in improving the understanding of the mechanisms that allow photosynthetic organisms to respond to the fluctuating conditions of the natural environments These fluctuations can be substantial, occurring on different timescales, ranging from the short term (in the sub-second range) to the long term (e.g. seasonal or even slower). Given the variety of adaptive strategies utilised by photosynthetic organism to thrive in specific habitats, the response mechanisms that have evolved to deal with environmental fluctuations are expected to be as diverse and complex.

The papers collected in this Research Topic reflect the variety of approaches employed to address the mechanism of response of photosynthetic organisms to fluctuating light conditions.

Gjindali et al. review both experimental and modelling approaches to address and describe short- and long-term responses to light fluctuations in land plants. A second review by Wang et al. discusses the limitations occurring under very dim light, in some species of vascular plants which generally are considered to have adapted to higher photon fluxes.

Moreover, Ye et al. demonstrate the benefits of mathematical descriptions that do not rely on asymptotic extrapolations to describe the light-saturation curves of net photosynthesis, carbon and oxygen fixation in land plants. Hommel et al. present a comparative study of short-term fluctuation and rapid responses of the photosynthetic apparatus to light-dark and spectral transitions, highlighting both similarities, as well as differences, in these responses in the model vascular plant *Arabidopsis thaliana*.

Other papers describe experimental investigations of different aspects responses to fluctuating light in plants, including species with agricultural potential. Tula et al. used the recombinant expression of flavodiiron (flv) proteins from *Synechocystis* sp.PCC6803, a recently discovered alternative electron sinks in cyanobacteria, algae and mosses (Ilík et al., 2017), to reduce the photoinhibition of Photosystem II under light fluctuation in Arabidopsis. The paper shows that Flv protein can be introduced in angiosperms and contribute in improving the photosynthetic electron transport efficiency. The study by Kaiser et al., performed in tomato, addresses the importance of stomatal conductance in ensuring a rapid establishment of photosynthetic fluxes upon dark-light transitions. Although Kaiser et al. present evidence that gas exchange can represent a limiting factor for the establishment of photosynthetic fluxes, and its increase appears therefore beneficial, it occurs at the expenses of water use efficiency. Therefore, there is a need to balance these two important factors which are often changing antagonistically. The importance of water use efficiency is also addressed in the paper by Dellero et al., showing that this factor is the most affected by the phenological variation in rapeseed leaves, despite a general trend of decreasing photosynthetic efficiency and carbon assimilation with age.

The study of Zhao et al. address the genetic factors controlling the production of anthocyanin in peach, particularly the promotion of their biosynthesis by UVA and UVB irradiance. Beside their role in fruit coloration, these pigments may also have an important screening effect to protect the organism from ultraviolet radiation. The generality of the photomorphogenic control is further demonstrated by Arabidopsis carrying the peach-derived regulatory elements.

The study by Ohkubo et al. compares different rice cultivars under fluctuating light and elevated CO_2_, showing a correlation between the best performing variety and the efficiency of nitrogen utilisation. Responses to fluctuating light are also a relevant factor in understanding the adaptation of plants to specific ecological niches, as shown by Kang et al. Their paper addresses the importance not only of light fluctuation, but also of the leaf temperature changes in response to fluctuating light; these act as synergistic factors, particularly in shade-tolerant plants. The acclimatory responses to light fluctuation can help us understand the ecological success of invasive species. The study of Mlinaric et al. showed that fast growth rate and invasive nature of the Japanese knotweed could be correlated with the increase ability to respond to light fluctuation, possibly linked to the ability of redirecting the photosynthetic electron transfer fluxes toward the cyclic electron transport pathway.

Further contributions to the Research Topic use unicellular cyanobacteria to investigate the acclimation to light changes at subcellular and molecular level. The study of Canonico et al. shows that controlled light oscillations have a significant effect on the average distribution of photosynthetic supercomplexes in defined membrane compartments. At the same time, the photosynthetic supercomplex distribution shows a broad variation on a cell-to-cell basis, which is suggested to represent a possible marker for phenotypic heterogeneity. The study of Bernat et al. addresses the effect of specific wavelengths on the composition of the photosynthetic apparatus of a picocyanobacterium, which displays a great plasticity. They report that cells illuminated with spectral ranges, particularly at the red edge of visible light (wavelengths longer than 680 nm), employ strategies previously considered exclusive to high light conditions.

On the whole, this Research Topic highlights the broad range of strategies that photosynthetic organisms may employ to adapt to ambient fluctuations, and how these strategies may not only depend on given taxa, but being highly heterogeneous down to leaf or cellular level.

## Author contributions

All authors listed have made a substantial, direct, and intellectual contribution to the work and approved it for publication.
